# Luteolin Inhibits Ischemia/Reperfusion-Induced Myocardial Injury in Rats via Downregulation of microRNA-208b-3p

**DOI:** 10.1371/journal.pone.0144877

**Published:** 2015-12-14

**Authors:** Chen Bian, Tongda Xu, Hong Zhu, Defeng Pan, Yang Liu, Yuanyuan Luo, Pei Wu, Dongye Li

**Affiliations:** 1 Institute of Cardiovascular Disease Research, Xuzhou Medical College, Xuzhou, Jiangsu, P. R. China; 2 Cardiology of Affiliated Hospital of Xuzhou Medical College, Xuzhou, Jiangsu, P. R. China; University of Cincinnati, College of Medicine, UNITED STATES

## Abstract

**Background:**

Luteolin (LUT), a kind of flavonoid which is extracted from a variety of diets, has been reported to convey protective effects of various diseases. Recent researches have suggested that LUT can carry out cardioprotective effects during ischemia/reperfusion (I/R). However, there have no reports on whether LUT can exert protective effects against myocardial I/R injury through the actions of specific microRNAs (miRs). The purpose of this study was to determine which miRs and target genes LUT exerted such function through.

**Methods:**

Expression of various miRs in perfused rat hearts was detected using a gene chip. Target genes were predicted with TargetScan, MiRDB and MiRanda. Anoxia/reoxygenation was used to simulate I/R. Cells were transfected by miR-208b-3p mimic, inhibitor and small interfering RNA of Ets1 (avian erythroblastosis virus E26 (v ets) oncogene homolog 1). MiR-208b-3p and Ets1 mRNA were quantified by real-time quantitative polymerase chain reaction. The percentage of apoptotic cells was detected by annexin V-fluorescein isothiocyanate/propidium iodide dyeing and flow cytometry. The protein expression levels of cleaved caspase-3, Bcl-2, Bax, and Ets1 were examined by western blot analysis. A luciferase reporter assay was used to verify the combination between miR-208b-3p and the 3’-untranslated region of Ets1.

**Results:**

LUT pretreatment reduced miR-208b-3p expression in myocardial tissue, as compared to the I/R group. And LUT decreased miR-208b-3p expression and apoptosis caused by I/R. However, overexpression of miR-208b-3p further aggravated the changes caused by I/R and blocked all the effects of LUT. Knockdown of miR-208b-3p expression also attenuated apoptosis, while knockdown of Ets1 promoted apoptosis. Further, the luciferase reporter assay showed that miR-208b-3p could inhibit Ets1 expression.

**Conclusion:**

LUT pretreatment conveys anti-apoptotic effects after myocardial I/R injury by decreasing miR-208b-3p and increasing Ets1 expression levels.

## Introduction

Coronary artery disease (CAD) poses a serious threat to public health and longevity worldwide. With the recent global prevalence of thrombolysis, percutaneous coronary intervention and coronary artery bypass surgery are commonly employed for treatment of CAD. Although effective reperfusion of the injured myocardium can minimize further damage, a series of adverse events often occur at the time of myocardial reperfusion, such as arrhythmia, myocardial dysfunction during systole and diastole, no reflow, and even sudden death [1]. Damage to myocardiocytes during myocardial ischemia-reperfusion (I/R) involves complex physiopathologic processes. Accordingly, the development of methods to alleviate or avoid myocardial I/R injury has become a primary focus of both basic and clinical research.

Several cellular and molecular biological events participate in the mechanisms of myocardial I/R injury, including oxidative stress, calcium overload, mitochondrial dysfunction, cell inflammation, and apoptosis. Of these, apoptosis is one of the most significant mechanisms of I/R injury, as a recent study demonstrated that myocardial I/R injury occurs in proportion to apoptosis [2]. Pre-treatment with various drugs is currently widely employed in both experimental research and clinical treatment. A large body of evidences has shown that flavonoid-rich herbs used in traditional Chinese medicine convey effective protection to injured myocardium during I/R [3].

Luteolin (LUT) is a flavonoid found in a variety of fruits, vegetables, and seeds, which conveys a variety of pharmacological properties, such as anti-inflammatory, anti-oxidation, and anti-tumor processes, as well as spasmolysis and immunoregulation [4, 5]. Recent evidence has suggested that LUT can protect myocardium during I/R [6]. However, precise mechanisms of LUT pre-treatment against myocardial I/R injury and identification of activated signaling pathways remain to be elucidated.

MicroRNAs (miRs) also play a significant role in protection against myocardial I/R injury [7]. The miRs are small (typically 18–25 nucleotides in length), non-coding, single-stranded RNAs involved in several cellular processes, including development, differentiation, and aging [8, 9]. They also participate in many post-transcriptional processes by binding to the 3’-untranslated region (3’-UTR) of target mRNAs to negatively regulate target gene expression [10]. The mode of inhibitory actions is dependent on the extent of binding of the miR to its target. Complete complementary binding leads to degragation of target mRNAs, while incomplete complementary binding results in translational inhibition of target proteins [11].

The results of many studies have affirmed that miRs have significant effects on myocardial I/R injury [12–14]. Nevertheless, to the best of our knowledge, no previous study has investigated whether LUT can alleviate myocardial I/R injury via regulation of the interactions of miRs with specific target genes. Therefore, in the present study, we employed gene chip technology, anoxia/reoxygenation (A/R) simulated I/R in H9c2 cells, real-time quantitative polymerase chain reaction (RT-qPCR), annexin V-fluorescein isothiocyanate (FITC)/propidium iodide (PI) (annexin V-FITC/PI, AV/PI) dyeing through flow cytometry, western blotting, and a luciferase reporter assay to explore how LUT attenuates apoptosis through specific miR-mediated regulation of target genes.

## Materials and Methods

### Study approval

The study protocol was approved by the Animal Ethics Committee of Xuzhou Medical College, Xuzhou, Jiangsu, China (permit no.: CMCACUC2013-04-107).

### Animals and reagents

Male Sprague Dawley (SD) rats, weighing 220–250 g, were housed in cages at Xuzhou Medical College at a temperature of 22°C under a 12-h light/dark cycle and randomly allocated into the following three groups: a control group, an I/R group, a LUT pretreatment (LUT + I/R) group. LUT (Fluka; purity > 98%; Sigma-Aldrich, Seelze, Germany) was dissolved in dimethyl sulfoxide (DMSO) and diluted with culture medium to a final concentration of 0.01% at the start of the experiments [15].

### Rat heart perfusion and further analysis

As described in our previous report [15], each male SD rat received an intraperitoneal injection of sodium heparin (1000 U/kg), and 10 min later, was anesthetized with sodium pentobarbital (150mg/kg). Then, the hearts were rapidly excised and mounted on a Langendorff perfusion apparatus. The different groups of rats were subjected to the following treatments. For the control group (n = 3), hearts were perfused with Krebs-Henseleit (KH) buffer for 200 min. For the I/R group (n = 3), hearts were perfused with KH solution for 50 min, then subjected to ischemia for 30 min and reperfused for 120 min with KH buffer. For the LUT pretreatment group (n = 3), hearts were perfused with KH solution for 20 min prior to LUT pretreatment with 40μM for 30 min, and then underwent I/R, as described above. Then, left ventricular systolic and diastolic function was monitored using a Biopac system (Biopac, USA) with a pressure sensor Millar transducer instrument. Heart rate (HR), left ventricular systolic pressure (LVSP), left ventricular end-diastolic pressure (LVEDP) and the rate in rise and fall of ventricular pressure (+dp/dt, -dp/dt) were recorded during the perfusion procedure. Afterward, the hearts were frozen and shipped to Kangcheng Biology Engineering Co., Ltd. (Shanghai, China) for gene chip and RT-qPCR analyzes.

### Cell culture

H9c2 cells were purchased from the Cell Bank of the Chinese Academy of Sciences (Shanghai, China) and maintained in Dulbecco's modified Eagle's medium (DMEM; Hyclone Laboratories, Inc., Logan, UT, USA) supplemented with 4.5 g/L of glucose, 10% (v/v) fetal bovine serum (Gibco-Invitrogen Australia Pty Ltd., Victoria, Australia), and 1% penicillin–streptomycin solution at 37°C under an atmosphere of 5% CO_2_.

### A/R simulated I/R protocol

The myocardiocytes were equilibrated in an incubator and then exposed to an ischemic buffer that contained the following (mM): 74 NaCl, 10 NaHCO_3_, 1.0 KH_2_PO_4_, 1.2 CaCl_2_, 1.2 MgCl_2_ 6H_2_O, 25 HEPES, 20 sodium lactate, and 16 KCl (adjusted to pH 6.2), which was previously equilibrated in an anoxia chamber (1% O_2_ and 5% CO_2_) at 37°C for 2h. The cells were incubated in the anoxia chamber during the entire 3-h simulated ischemic period. Ischemia buffer was then replaced with serum-free DMEM under normoxic conditions during the 2-h reperfusion process [16].

### Cell viability assay

To assess cellular viability, 1×10^3^ H9c2 cells were added to the wells of a 96-well plate. The different groups were pretreated with various concentration of LUT (2, 4, 8, 16, 32, 64μM). Each group occupied three wells on the plate. Viability of myocardiocytes was detected using the CCK-8 assay after A/R. Briefly, in the absence of light, CCK-8 reagent (10 ul) was added to each welland the plates were incubated at 37°C under an atmosphere of 5% CO_2_ for 1–4 h. Then, the optical densities were detected using a microplate reader (BioTek Instruments, Inc., Winooski, VT, USA).

### Transient transfection with oligonucleotides

H9c2 cells were plated in a 6-well plate at a concentration of 3×10^5^ cells per well and transfected with a specific miR-208b-3p mimic or a duplex RNA inhibitor (Shanghai GenePharma Co., Ltd., Shanghai, China), of which the sense sequence was the same as that of miR-208b-3p or complementary to miR-208b-3p, to overexpress or knockdown miR-208b-3p. Thereafter, the cells were transfected with small interfering RNA (siRNA) (Shanghai GenePharma Co., Ltd.) to knockdown Ets1 (avian erythroblastosis virus E26 (v ets) oncogene homolog 1). All transfections were carried out after serum starvation for 12h to achieve cell cycle synchronization [17]. After transfection for 6h, the medium was replaced with fresh medium containing serum and then the cells were further cultured for an additional 24h. Then, the cultures were serum-deprived for 12h to achieve synchronization and exposed to hypoxia for 3h and reoxygenation for 2h. All transfections were performed using Lipofectamine2000 transfection reagent (Invitrogen Corporation, Carlsbad, CA, USA), in accordance with the manufacturer’s protocol. Non-targeting sequences were used as negative controls.

### AV/PI dual staining for detection of apoptotic cells

H9c2 cells were plated in a 6-well plate at a concentration of 3×10^5^ cells per well. After the different groups were subjected to the various processing methods, the percentage of apoptotic cells was detected using the Annexin V-FITC/PI Apoptosis Detection Kit (Nanjing KeyGen Biotech Co., Ltd., Nanjing, China). H9c2 cells were harvested by centrifugation at 2,000 rpm for 5 min, washed with phosphate-buffered saline, and centrifuged again at 2,000 rpm for 5 min, followed by the addition of binding buffer (500 μl) to disperse the cells and the addition of annexin V-FITC (5 μl), and PI (5 μl). Next, the cell cultures were incubated at room temperature in the dark for 5–15 min and analyzed at an excitation wavelength of 488 nm and emission wavelength of 530 nm using a BD FACSCanto II flow cytometer (BD Biosciences, San Jose, CA, USA) equipped with a FITC signal detector (FL-1) and by PI staining using a phycoerythrin emission signal detector (FL-2). Fluorescent intensity was acquired over 10,000 events. Since the annexin V positive and PI negative populations represented early apoptotic cells, and the populations of both annexin V and PI positive represented the summation of late apoptotic cells and necrotic cells, annexin V positive and PI negative populations were usually chosen as the index of apoptotic cells [18–20].

### RT-qPCR analysis

Total RNA (1 μg) was extracted with Trizol Reagent (Invitrogen Corporation) and reverse-transcribed using Moloney murine leukemia virus reverse transcriptase with oligo-dTs into complementary DNA. RT-qPCR was performed using an ABI7500 real-time DNA detection system (Applied Biosystems, Waltham, MA, USA) with SYBR Green Master Mix (TIANGEN Biotech (Beijing) Co., Ltd., Beijing, China). The primers for miR-208b-3p, U6, Ets1 and β-actin were designed by Shanghai Generay Biotech Co., Ltd. (Shanghai, China). All samples were analyzed in triplicate. The relative expression levels of miR-208b-3p and its target gene, Ets1, were detected by the standard curve method, and normalized against U6 and β-actin, respectively.

### Plasmid construction and analysis of luciferase activity

Ets1 was predicted as one of the putative target genes of miR-208b-3p by TargetScan (www.targetscan.org), MiRDB (http://mirdb.org/miRDB/), and MiRanda (http://cbio.mskcc.org/miRNA2003/miranda.html). To examine whether miR-208b-3p regulates the expression of Ets1, a dual luciferase GP-Check2 reporter plasmid (Shanghai GenePharma Co., Ltd.) was used to generate a reporter plasmid harboring the 3’-UTR of Ets1. The Ets1 3’-UTR containing the binding sequence CGTCTTA with miR-208b-3p was amplified from rat genomic DNA by PCR and cloned into the sequence between XhoI and NotI restriction sites of plasmid GP-Check2, which was considered the wild type (WT) plasmid. In addition, the GP-Check2 plasmid, a mutant type (MUT) plasmid containing a mutated targeting fragment GCAGAAT, served as the transfection control. Briefly, H9c2 cells were co-transfected with the WT/MUT GP-Check2 vector containing Ets1 3’-UTR and miR-208b-3p mimic/inhibitor using Lipofectamine 2000, while co-transfection with a non-targeting RNA sequence served as a negative control. The cells were harvested 24h after transfection, then luciferase activity was measured using a dual luciferase reporter assay kit (Promega Corporation, Madison, WI, USA) on a FB 12 Single Tube Luminometer (Berthold Detection Systems GmbH, Pforzheim, German).

### Western Blotting analysis

The H9c2 cells were collected, lysed with RIPA lysis buffer (Beyotime Institute of Biotechnology, Haimen, China) supplemented with phenylmethylsulfonyl fluoride, and then cooled on ice for 30 min. After centrifugation at 12,000 rpm for 15 min at 4°C, the supernatant was collected and protein concentrations were measured using a modified Bradford assay (Bio-Rad Laboratories, Inc., Hercules, CA, USA). Protein samples (20μg) were separated by sodium dodecyl sulfate–polyacrylamide gel electrophoresis on 8%–15% gels and then transferred to polyvinylidene difluoride membranes. After blocking with 5% bovine serum albuminin Tris-buffered saline containing 0.1% Tween 20 at room temperature for 3h, the membranes were immuno-blotted with primary antibodies against caspase-3 (dilution, 1:1000; Cell Signaling Technology, Beverly, MA, USA), Bcl-2 (dilution, 1:500; Santa Cruz Biotechnology, Inc., Santa Cruz, CA, USA), Bax (dilution, 1:500; Santa Cruz Biotechnology, Inc.), Ets1 (dilution, 1:400; Wuhan Boster Biological Technology, Ltd., Wuhan, China), and β-actin (dilution, 1:2000; Beijing Zhongshan Golden Bridge Biotechnology Co., Ltd., Beijing, China) overnight at 4°C. This step was followed by incubation with peroxidase-conjugated secondary antibodies (dilution, 1:10000; Beijing Zhongshan Golden Bridge Biotechnology Co., Ltd.) at room temperature for 1h. Protein bands were visualized by enhanced chemiluminescence reagent (Pierce Biotechnologies, Thermo Fisher Scientific, Waltham, MA, USA), according to the manufacturer’s protocol, and then imaged using Image J 3.0 software (http://imagej.nih.gov/ij/).

### Statistical analysis

Graph Pad Prism 5 software was used for all statistical analyses. Data are presented as means ± standard error of the mean. Comparisons among various groups were performed using one-way and two-way analysis of variance. The Student's *t*-test was employed (two sided, assuming similar variances) when there were only two groups. A probability (p) value of < 0.05 was considered statistically significant. All experiments were performed at least three times.

## Results

### Hemodynamic effects of luteolin on isolated rat hearts subjected to I/R

**Fig 1:**
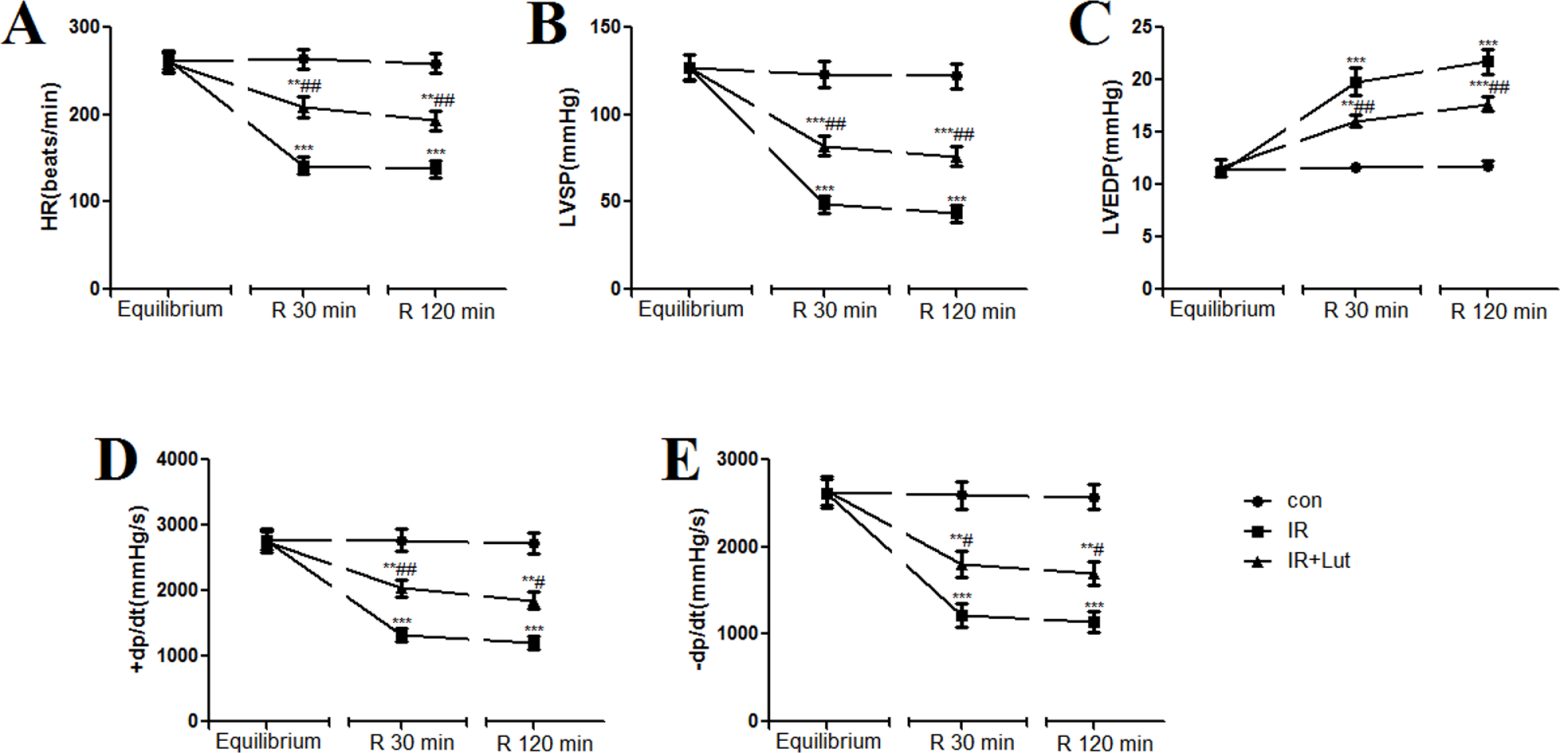
Effects of luteolin on various parameters of hemodynamic measurements during a perfusion period in isolated rat hearts. I/R decreased HR (A), LVSP (B), +dp/dt (D) and -dp/dt (E), and increased LVEDP (C), ***p<0.001 as compared to the control group. While LUT pre-treatment increased HR (A), LVSP (B), +dp/dt (D) and -dp/dt (E), and decreased LVEDP (C), ##*p*<0.01, #*p*<0.05 as compared to the I/R group. Equilibrium indicated baseline, R 30min indicated reperfusion 30 min, and R 120 min indicated reperfusion 120 min.

The hemodynamic results showed that there were no significant differences among the groups at the beginning of perfusion. As compared to the control group, I/R decreased HR, LVSP, +dp/dt and -dp/dt, and increased LVEDP. While LUT pre-treatment increased HR, LVSP, +dp/dt and -dp/dt, and decreased LVEDP as compared to the I/R group (Fig 1).

### Expression of miRs in isolated rat hearts after I/R injury following LUT pre-treatment

**Fig 2:**
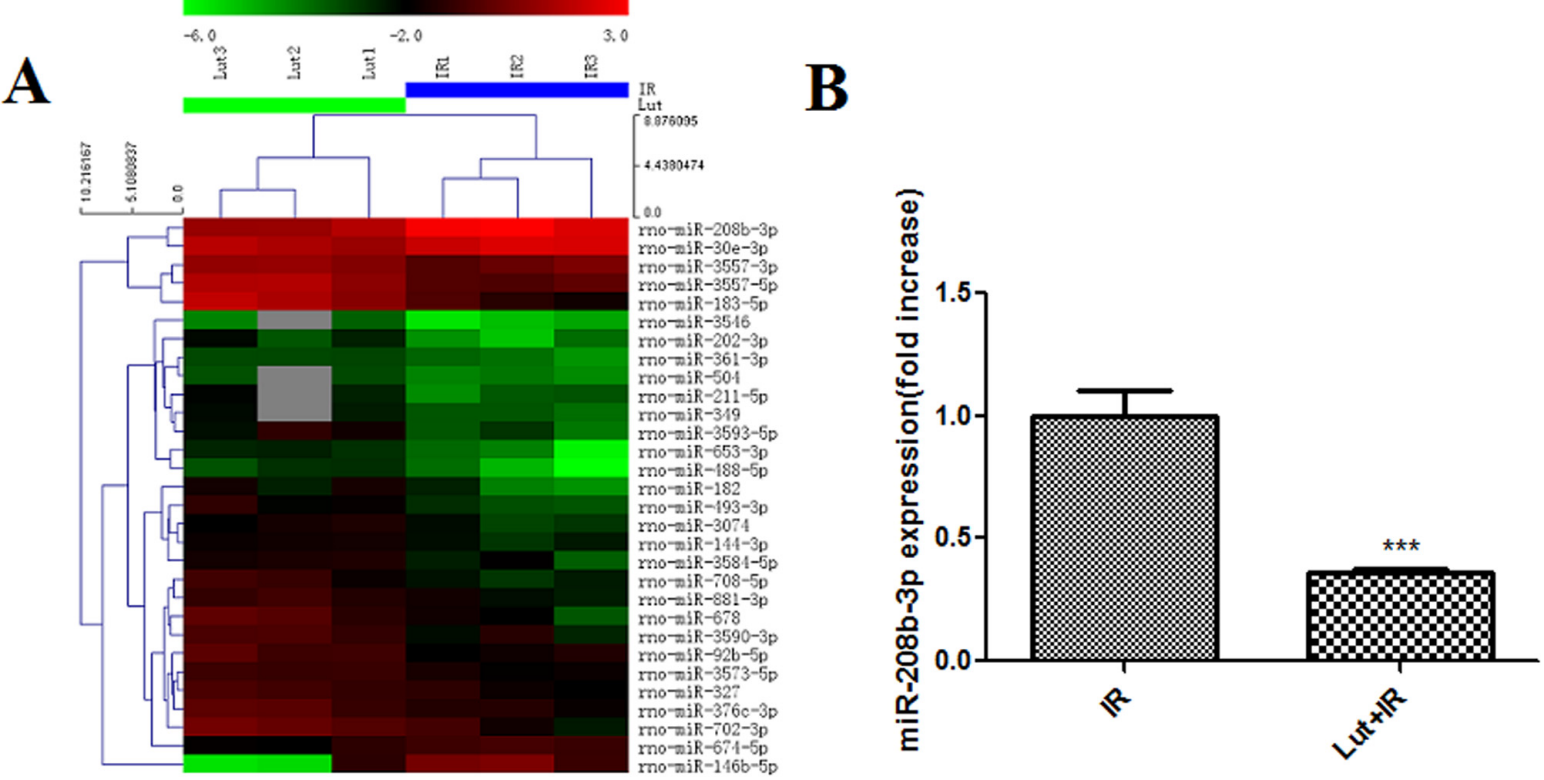
MiRs expression level in perfusion rat hearts by gene chip and RT qPCR. A. Gene chip detected the microRNAs relating LUT versus IR. B. RT-qPCR detected miR-208b-3p expression in perfusion myocardial tissue. LUT pretreatment could down-regulate miR-208b-3p expression, ***p<0.001 as compared with IR group.

The results of gene chip assay showed that LUT pre-treatment down-regulated the expression levels of four miRs and up-regulated the expression levels of 26 miRs, as compared to the I/R group (Fig 2A). The results of RT-qPCR analysis showed that LUT pre-treatment reduced miR-208b-3p expression in the myocardiocytes, as compared to I/R group (0.37 ± 0.04, *p* < 0.001) (Fig 2B).

### LUT pre-treatment protects H9c2 cells against A/R injury

**Fig 3:**
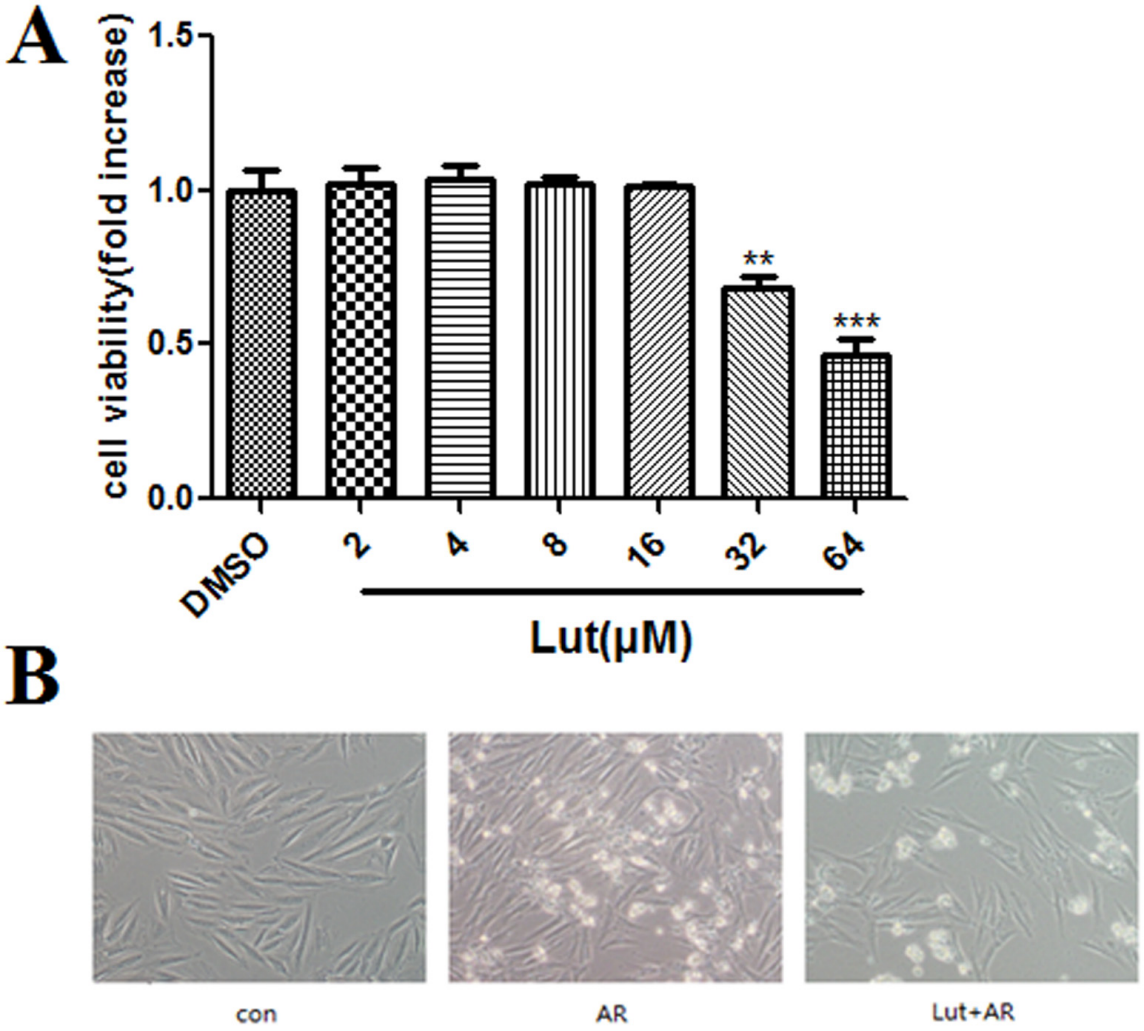
Cell viability by CCK-8 assay under LUT pretreatment and the morphology of H9c2 cells. A. CCK-8 assay detected the toxicity of LUT on H9c2 myocardiocytes. Compared to DMSO group, 32 and 64μM of LUT reduced the cell viability (**p<0.01, ***p<0.001), 16μM of LUT was chosen for the further experiments. B. The morphology of H9c2 cells in control, AR and LUT+AR group.

The results of the CCK-8 assay showed that, as compared to the DMSO control group, 32 and 64 μM of LUT decreased cell viability to 0.69 ± 0.04 (*p* < 0.01) and 0.47 ± 0.05 (*p* < 0.001), respectively. However, 2, 4, 8 and 16 μM of LUT showed no detectable toxicity, thus, we chose 16.0 μM of LUT for the following experiments (Fig 3A).

As compared to the control group, in which cells had fusiform and regular morphologies, A/R injury caused an irregular morphology with many dead cells, whereas LUT pre-treatment was found to reverse the process caused by A/R, as cell membranes were complete and nuclei were located in the middle of the cells (Fig 3B).

**Fig 4:**
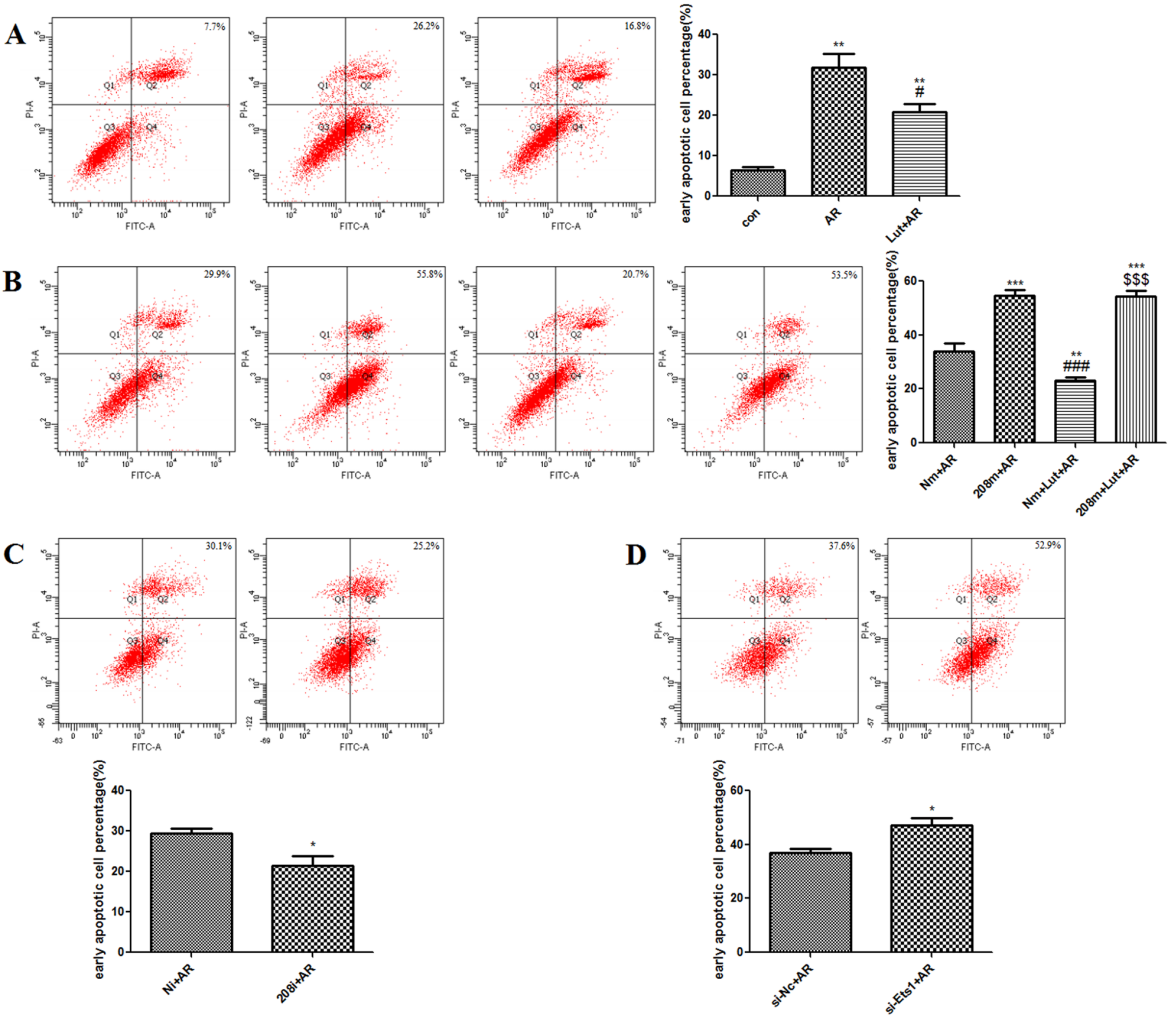
AV/PI detected the early apoptotic cell percentage of H9c2 cells. **A.** AR significantly increased the early apoptotic cell percentage of H9c2 cells compared with control group (**p<0.01). However, LUT pretreatment significantly decreased the apoptosis rate compared to AR group (#*p*<0.05). B. Overexpression of miR-208b-3p under AR significantly increased the early apoptotic cell percentage of H9c2 cells compared with AR group (***p<0.001). However, LUT pretreatment had no statistical significance compared to 208m+AR group (*p*>0.05). C. Knocking down miR-208b-3p expression under AR decreased the early apoptotic cell percentage of H9c2 cells compared with Ni+AR group (*p<0.05). D. Knocking down Ets1 expression under AR increased the early apoptotic cell percentage of H9c2 cells compared with si-Nc+AR group (*p<0.05).

AV/PI dual staining showed that, as compared to the control group, A/R injury increased the percentage of H9c2 cells in the early phase of apoptosis (31.93 ± 3.26% vs. 6.47 ± 0.75%, *p* < 0.01). LUT pre-treatment significantly decreased the percentage of H9c2 cells in the early phase of apoptosis, as compared to the AR group (20.87 ± 2.03%, *p* < 0.05) (Fig 4A).

### LUT pre-treatment down-regulates miR-208b-3p during A/R

The RT-qPCR results showed that, as compared to the control group, A/R injury increased miR-208b-3p expression (4.37 ± 0.12, *p* < 0.01), while LUT pre-treatment reduced miR-208b-3p expression in the H9c2 cells, as compared to the AR group (2.23 ± 0.43, *p* < 0.01) (Fig 7A).

### Efficiency of oligonucleotide transfection

**Fig 5:**
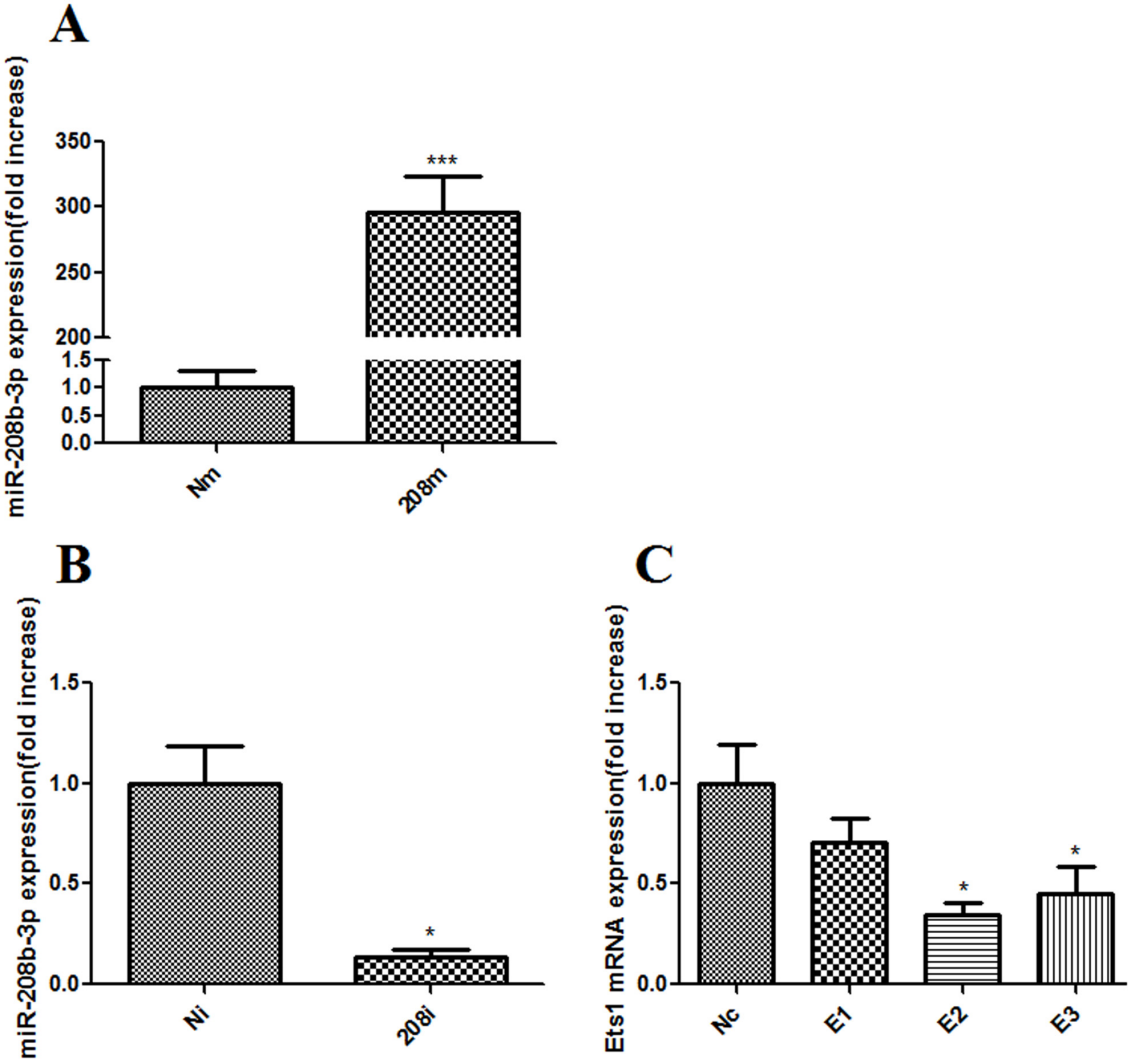
Efficiency of oligonucleotides transfection detected by RT-qPCR. A. RT-qPCR verified the overexpression of miR-208b-3p by transfecting miR-208b-3p mimic (208m group) in H9c2 cells, ***p<0.001 as compared with Nm group. B. Transfecting miR-208b-3p inhibitor (208i group) could significantly knock-down miR-208b-3p expression in H9c2 cells, ***p<0.001 as compared with Ni group. C. The mRNA expression of Ets1 after knocking-down it by si-RNAs. Compared to Nc group, E2 and E3 reduced Ets1 mRNA expression by 66%, 55% (*p<0.05), E2 was chosen for the further experiments.

Transfection with the miR-208b-3p mimic significantly increasedmiR-208b-3p expression levels by 296.10 ± 27.63-fold versus the Nc mimic group (*p* < 0.001) (Fig 5A), and transfection with the miR-208b-3p inhibitor decreased the level of miR-208b-3p in cultured H9c2 cells by 0.14 ± 0.04-fold versus the Nc inhibitor group (*p* < 0.05) (Fig 5B). After transfection with three Ets1-siRNA sequences, respectively, the second sequence most significantly decreased Ets1 mRNA expression (0.34 ± 0.06, *p* < 0.05 vs. Nc) (Fig 5C). Therefore, this sequence was chosen for the following experiments.

### After LUT pre-treatment and transfection with the miR-208b-3p mimic/inhibitor, Ets1-siRNA was found to affect the percentage of H9c2 cells in the early phase of apoptosis

AV/PI dual staining revealed that after transfection with the miR-208b-3p mimic, the percentage of H9c2 cells in the early phase of apoptosis increased (54.60 ± 2.05% vs. 33.93 ± 3.04%, *p* < 0.001). However, LUT pre-treatment had no effect on the A/R-induced changes following transfection with the miR-208b-3p mimic (*p* > 0.05) (Fig 4B). On the other hand, transfection with the miR-208b-3p inhibitor was found to decrease the percentage of H9c2 cells in the early phase of apoptosis (21.47 ± 2.33% vs. 29.37 ± 1.32%, *p* < 0.05) (Fig 4C). Transfection with Ets1-siRNA increased the percentage of H9c2 cells in the early phase of apoptosis (47.03 ± 2.93% vs. 36.93 ± 1.37%, *p* < 0.05) (Fig 4D).

### Results of western blot analysis

**Fig 6:**
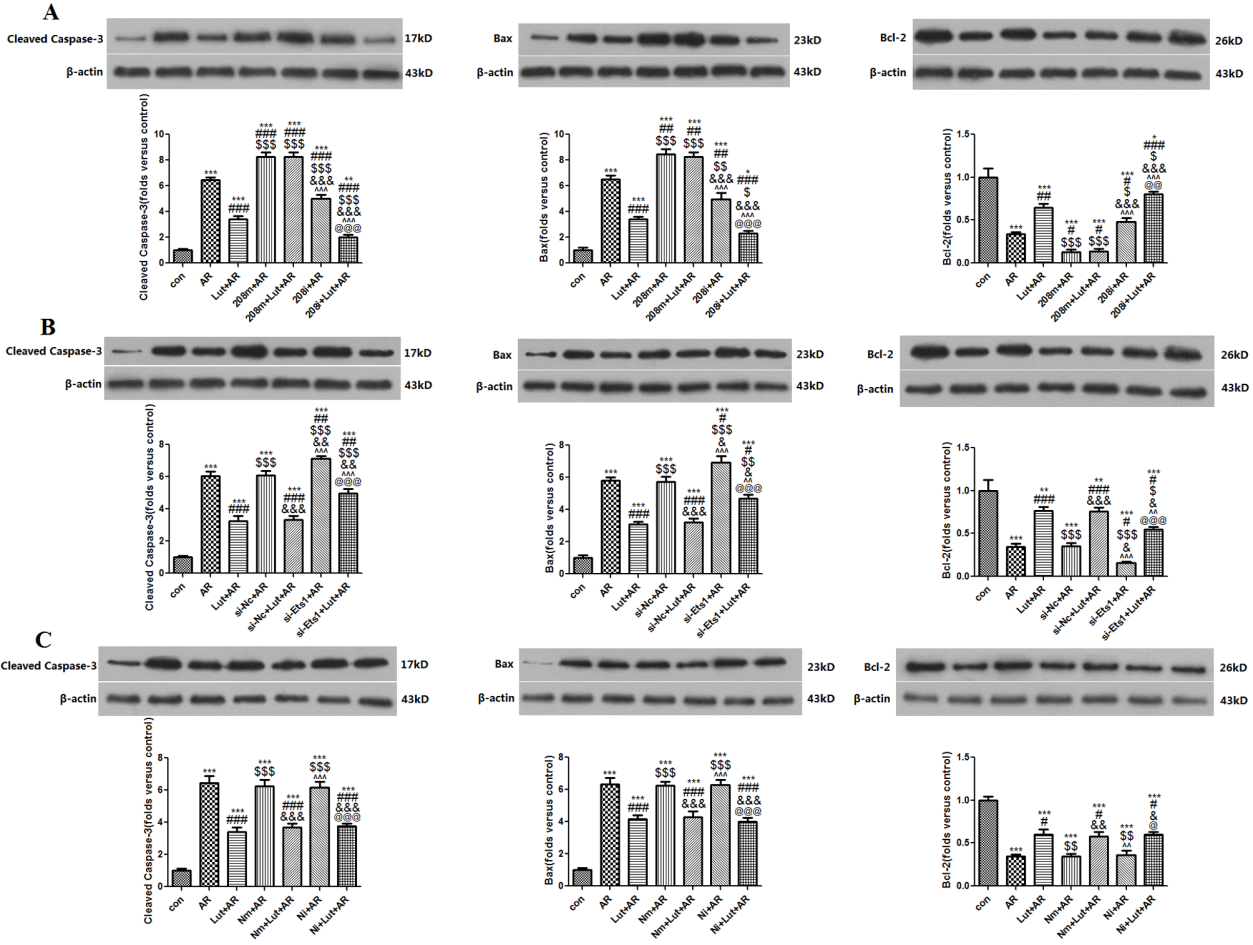
Apoptosis protein detected by Western Blotting assay. A. Cleaved caspase-3 and Bax was increased after A/R injury, but reduced by LUT pretreatment in H9c2 cells, ***p<0.001 as compared with control group, ###*p*<0.001 as compared with AR group; Over-expressing miR-208b-3p increased cleaved caspase-3 and Bax, ###*p*<0.001, ##*p*<0.01 as compared with AR group, and abolished the effects of LUT on cleaved caspase-3 and Bax (*p*>0.05). Knocking down miR-208b-3p decreased cleaved caspase-3 and Bax, ###*p*<0.001, ##*p*<0.01 as compared with AR group. While Bcl-2 was decreased after A/R injury, but increased by LUT pretreatment in H9c2 cells, ***p<0.001 as compared with control group, ##*p*<0.01 as compared with AR group; Over-expressing miR-208b-3p decreased Bcl-2, #*p*<0.05 as compared with AR group, and abolished the effects of LUT on Bcl-2 (*p*>0.05). Knocking down miR-208b-3p increased Bcl-2, #*p*<0.05 as compared with AR group. B. Cleaved caspase-3 and Bax were increased after knocking down Ets1, ##*p*<0.01, #*p*<0.05 as compared with AR group; While Bcl-2 was decreased after knocking down Ets1, #*p*<0.05 as compared with AR group. C. Transfecting Nc mimic/inhibitor had no statistical significance on the expression of cleaved caspase-3, Bax and Bcl-2.

**Fig 7:**
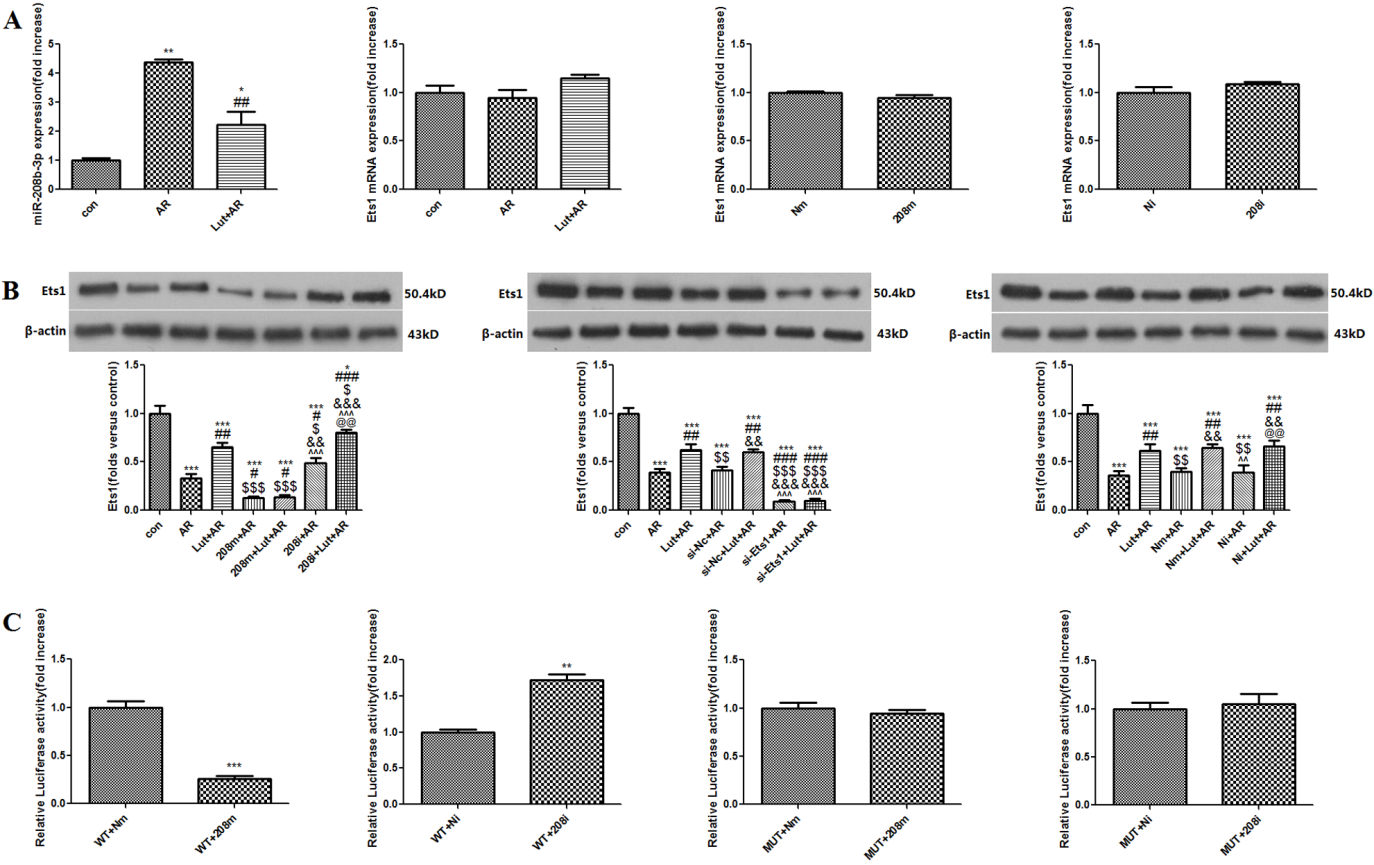
Ets1 was a target of miR-208b-3p. A. MiR-208b-3p was detected to be up-regulated under A/R injury, LUT pretreatment could down-regulate it in H9c2 cells, **p<0.01, *p<0.05 as compared with control group; ##*p*<0.01 as compared with AR group. Ets1 mRNA was detected to have no statistical significance under A/R injury and LUT pretreatment (*p*>0.05). Ets1 mRNA was detected to have no statistical significance under miR-208b-3p mimic/inhibitor transfecting (*p*>0.05). B. Ets1 was decreased after A/R injury, but increased by LUT pretreatment in H9c2 cells, ***p<0.001 as compared with control group, ##*p*<0.01 as compared with AR group; Over-expressing miR-208b-3p decreased Ets1, #*p*<0.05 as compared with AR group, and abolished the effects of LUT on Ets1 (*p*>0.05). Knocking down miR-208b-3p increased Ets1, #*p*<0.05 as compared with AR group. And Ets1 was decreased after transfecting Ets1-siRNA, ###*p*<0.001 as compared with AR group, and abolished the effects of LUT on Ets1 (*p*>0.05). C. By transfecting of the reporter plasmid with (WT group) or without (MUT group) the putative miR-208b-3p binding site, the luciferase activity decreased after miR-208b-3p overexpression and increased after miR-208b-3p knock-down in H9c2 cells; ***p<0.001 as compared with WT+Nm group, **p<0.01 as compared with WT+Ni group. In addition, mutating the predicted binding site of miR-208b-3p (MUT+208m, MUT+208i group) abolished the process mentioned above (*p*>0.05).

The results of western blot analysis demonstrated that A/R injury increased cleaved caspase-3 and Bax protein levels, and decreased Bcl-2 protein levels (*p* < 0.001 vs. the control group). As compared to the AR group, cleaved caspase-3 and Bax protein levels of the LUT+AR group were decreased, while Bcl-2 protein levels were increased (*p* < 0.01 and < 0.001, respectively vs. the AR group) and cleaved caspase-3 and Bax protein levels of the 208m+AR group were increased, while Bcl-2 protein levels were decreased (*p* < 0.05, < 0.01, and < 0.001, respectively vs. the AR group). Also, cleaved caspase-3 and Bax protein levels in the 208i+AR group were decreased, while Bcl-2 protein levels were increased (*p* < 0.05, < 0.01, and < 0.001, respectively vs. the AR group). Nevertheless, there were no statistically significant differences in the expression levels of these apoptotic proteins between the 208m+AR and 208m+LUT+AR groups (*p* > 0.05) (Fig 6A). Cleaved caspase-3 and Bax protein levels of the si-Ets1+AR group were increased, while Bcl-2 protein levels were decreased (*p* < 0.05 and < 0.01, respectively vs. the AR group) (Fig 6B). A/R was found to decrease Ets1 protein levels (*p* < 0.001 vs. the control group). As compared to the AR group, Ets1 protein levels in the LUT+AR and 208i+AR groups were increased (*p* < 0.05 and < 0.01, respectively), Ets1 protein levels of the 208m+AR and si-Ets1+AR groups were decreased (*p* < 0.05), Ets1 protein levels of 208i+AR group were increased (p < 0.05), and Ets1 protein levels of the si-Ets1+AR group were decreased (*p* < 0.05). However, there were still no statistically significant differences in Ets1 protein expression levels between the 208m+AR and 208m+LUT+AR groups (*p* > 0.05) or between the si-Ets1+AR and si-Ets1+LUT+AR groups (p > 0.05) (Fig 7B).

### Ets1 is a target gene of miR-208b-3p

One miR down-regulated its target gene by connecting to the 3’-UTR. Therefore, we cloned the 3’-UTR of Ets1 downstream of a luciferase gene and mutated the predicted binding site of miR-208b-3p in the 3’UTR of Ets1 as a mutative plasmid. Overexpression of miR-208b-3p caused a significant decrease in luciferase activity (*p* < 0.001), and transfection with the miR-208b-3p inhibitor increased luciferase activity (*p* < 0.01), suggesting that Ets1 is a target gene of miR-208b-3p. In addition, of the predicted miR-208b-3p binding site abolished this process (*p* > 0.05) (Fig 7C).

Transfection with the miR-208b-3p mimic decreased Ets1 protein levels, while transfection with the miR-208b-3p inhibitor increased Ets1 protein levels (*p* < 0.05, 208m+AR and 208i+AR groups vs. AR group) (Fig 7B). However, there were no significant differences in Ets1 mRNA levels after A/R and LUT pre-treatment (p > 0.05 vs. control group). Also, overexpression or knockdown of miR-208b-3p had no significant effect on Ets1 mRNA levels (p > 0.05 vs. the Nm and Ni groups) (Fig 7A).

## Discussion

In recent years, the mechanism of I/R has been investigated intensively. It is well known that apoptosis is closely correlated to I/R injury and is involved in the I/R process [2, 16, 21]. A large quantity of evidence has revealed increased apoptosis in many organs and tissues after I/R [22] and shown that the regulation of anti-apoptosis is relative to the regulation of many genes, such as the Bcl-2 family, caspase family, p53, and nuclear factor-κB. Apoptosis of myocardiocytes is the critical cellular event of myocardial I/R injury. An increasing number of studies have focused on how to alleviate apoptosis of myocardiocytes.

The results of several studies have shown that the pre-treatment and post-treatment with Chinese medicines rich in flavonoids convey safe and effective protection to myocardiocytes following I/R injury [23, 24]. Also, Hertog et al. [25] reported that routine intake of flavonoids, such as LUT, could reduce the incidence of myocardial infarction. Other epidemiological data derived from population health surveys showed that diets rich in LUT can decrease the risk of acute myocardial infarction [3]. Previous studies by our group also verified that LUT activates the PI3K/Akt and p-ERK pathways, and inhibits the JNK pathway by regulating relative protein expression to exert anti-apoptotic effects and improving myocardium contraction [15, 26, 27].

It is well-known that miRs have significant effects on the regulation of apoptosis of myocardiocytes following I/R injury. However, it remains unclear as to whether LUT conveys a protective effect to the myocardium after I/R through regulation of some miRs, as there are no previous reports of the ability of LUT to alleviate damage to the myocardium after I/R injury via certain miRs that regulate specific target genes.

Currently, the identification of other miRs is ongoing. It is well-known that specific gene expression and miRs regulation participates in cell apoptosis. An increasing number of researches have focused on changes in expression levels of miRs and the identification of concrete mechanisms in apoptosis. Other anti-apoptotic miRs have also been recently identified, including miR-21 [12], miR-25 [28], and miR-378 [17]. On the other hand, a few miRs have been found to promote apoptosis of myocardiocytes, such as miR-26 [29], miR-34 [30], and miR-92 [31].

In the present study, we detected the function of the isolated hearts, and found that hemodynamic effects were improved following LUT pre-treatment, as compared to the I/R group. Then we used gene chip technology to detect expression of various miRs. The results verified significant changes in the expression levels of many miRs following LUT pre-treatment. As compared to the I/R group, LUT pre-treatment up-regulated the expression levels of 26 miRs and down-regulated that of four. Among the four down-regulated miRs, changes in miR-146b-5p and miR-208b-3p expression levels were obvious. The literatures of miR-208 on cardiovascular diseases were more than miR-146. Studies have reported that miR-208 is specific to myocardiocytes, at least to some extent [32, 33]. In recent years, studies of miR-208 mainly concentrated on plasma biomarkers [33], acute myocardial infarction [34], heart failure [35], and ventricular remodeling [36]. In addition, the roles of miR-208 in heart diseases, such as myocardial hypertrophy [37] and myocardial fibrosis [38], have also been reported. The results of a large-scale clinical trial demonstrated that miR-208 could be detected in the blood of patients after early acute myocardial infarction [33]. However, there have no reports on the relationship between miR-208 and apoptosis of myocardiocytes following I/R injury.

The complicated mechanism between miR-208 and myocardial I/R injury needs to be further explored. To better elucidate the mechanism between miR-208 and myocardial I/R injury, we performed the experiments described in this report according to some of the methods reported by Fang et al. [17], who used a miR microarray to analyze an in vivo rat model of 4-h myocardial ischemia without reperfusion and found that miR-378 overexpression conveyed a protective role to cardiomyocytes following ischemic injury. While our study employed an ex vivo rat myocardium model of I/R injury using the perfusion method described in our previous report, which showed that LUT improved the contractile function of cardiomyocytes after I/R injury through an ERK1/2-PP1a-PLB-SERCA2a-mediated mechanism independent of the JNK signaling pathway [15]. Our present results showed that miR-208b-3p expression levels were up-regulated in H9c2 cells, as compared to the control group. After overexpression and knockdown miR-208b-3p, A/R and LUT pre-treatment, AV/PI dyeing was used to determine the percentage of H9c2 cells in the early phase of apoptosis by flow cytometry. Also, western blot analysis was conducted to detect expression levels of the apoptotic proteins caspase-3, Bcl-2 and Bax. We found that down-regulation of miR-208b-3p decreased the percentage of H9c2 cells in the early phase of apoptosis and inhibited expression of cleaved caspase-3 and Bax, while it increased Bcl-2 expression. LUT was found to reduce miR-208b-3p expression, thereby attenuating apoptosis. Thus, we concluded that overexpression of miR-208b-3p aggravates apoptosis and knockdown of miR-208b-3p alleviates apoptosis. LUT pre-treatment can decrease the extent of A/R-induced apoptosis. We also found that miR-208b-3p overexpression abolished the protect effects of LUT. Accordingly, the anti-apoptotic effect of LUT is dependent on knockdown of miR-208b-3p. However, the mechanisms regarding how LUT regulates the expression of miR-208b-3p remain unclear. There are various pattern of miRs regulation, for instance, some certain factors may change specific miRs expression by epigenetic modification [39], stimulating one or more links of a signal pathway [40], or regulating some transcription factors [41]. We inferred that LUT might also reduce the production of miR-208b-3p or induce miR-208b-3p degradation through epigenetic modification, signal pathway or regulating specific transcription factors.

Each miR can regulate the expression of different target genes in a particular signaling pathway. Likewise, each gene may also be a target of different miRs. Thus, miRs and their targets make up a complex regulation network, which controls vital cellular activities [10]. Two mechanisms of miRs affecting the expression of their target genes have already been identified. When a miR is incompletely complementary paired with the 3’-UTR of its target mRNA, expression of the target gene is decreased at the protein level, but this has no effect on the stability of the target mRNA. When a miR completely complementary pairs with a target sequence, the miR will have an interference effect and cause degradation of the target mRNA, thereby mediating gene silencing at the mRNA level. This type of action is similar to that of siRNA, although siRNA acts on the coding region of double-stranded RNA [11]. In short, the extent complementary of a miR and its target mRNA determine the regulation mode of the miR.

A major finding of this study was that miR-208b-3p expression significantly reduced Ets1 protein levels, but had no significant effect on mRNA levels, suggesting that miR-208b-3p regulates Ets1 expression at the post-transcription level. Since one miR can regulate the expression of its target gene through both transcription and post-transcription mechanisms, we inferred that miR-208b-3p incompletely complementary binds to the 3’-UTR of Ets1, which only inhibits translation after transcription and prevents synthesis of the protein, while not impacting the stability of the mRNA. It is significant in such a process that down-regulation of miR-208b-3p results in an increase in Ets1 expression. This result provides a possible basis of miR-208b-3p regulation of Ets1 expression and an ultimate effect on cellular activities.

Ets1 is a member of the largest family of transcription factors and is unique to metazoans. The genes of this family are thought to have been transferred from the E26 leukemia virus [42]. The members of this family are thought to participate in the regulation of cellular growth and development, as well as cancer progression [43–45]. The ETS family participates in many cell regulation activities, including differentiation, cell cycle regulation, migration, proliferation, apoptosis, and angiogenesis [46]. Ets1 is the first identified member of the ETS family. There have been relatively few reports of the roll of Ets1 on apoptosis of myocardiocytes. Wang et al. [47] found that hyperglycemia induced apoptosis of primary cardiomyocytes in the rat through ERK-dependent activation of Ets1. However, the results of the present study demonstrated that Ets1 regulated cell apoptosis through increasing apoptotic protein Bax and Caspase-3 expression meanwhile decreasing anti-apoptotic protein Bcl-2 expression. Namely, Ets1 knockdown aggravated I/R-induced apoptosis of H9c2 cells. This discrepancy may be due to differences in cell types and experimental conditions employed in these studies.

In summary, the findings of the present study further elucidated this phenomenon and identified a mechanism through which LUT alleviates myocardial I/R injury via the regulation of specific miRs for the first time. We can conclude that LUT attenuates apoptosis after I/R injury through down-regulation of miR-208b-3p and up-regulation of Ets1 expression. The results of this study provide theoretical and experimental foundations for further research, and offer a new therapeutic target for future clinical use.
